# The microbial carbonate factory of Hamelin Pool, Shark Bay, Western Australia

**DOI:** 10.1038/s41598-022-16651-z

**Published:** 2022-07-28

**Authors:** Erica P. Suosaari, R. Pamela Reid, Christophe Mercadier, Brooke E. Vitek, Amanda M. Oehlert, John F. Stolz, Paige E. Giusfredi, Gregor P. Eberli

**Affiliations:** 1 Department of Mineral Sciences, National Museum of Natural History, Smithsonian Institution, Washington, DC 20560 USA; 2 Rosenstiel School of Marine and Atmospheric Science, University of Miami, Miami, FL 33149 USA; 3 Bush Heritage Australia, 395 Collins St., Melbourne, VIC 3000 Australia; 4 Shell International, Colombes, France; 5 Department of Biological Sciences, Duquesne University, Pittsburgh, PA 15282 USA

**Keywords:** Solid Earth sciences, Sedimentology

## Abstract

Microbialites and peloids are commonly associated throughout the geologic record. Proterozoic carbonate megafacies are composed predominantly of micritic and peloidal limestones often interbedded with stromatolitic textures. The association is also common throughout carbonate ramps and platforms during the Phanerozoic. Recent investigations reveal that Hamelin Pool, located in Shark Bay, Western Australia, is a microbial carbonate factory that provides a modern analog for the microbialite-micritic sediment facies associations that are so prevalent in the geologic record. Hamelin Pool contains the largest known living marine stromatolite system in the world. Although best known for the constructive microbial processes that lead to formation of these stromatolites, our comprehensive mapping has revealed that erosion and degradation of weakly lithified microbial mats in Hamelin Pool leads to the extensive production and accumulation of sand-sized micritic grains. Over 40 km^2^ of upper intertidal shoreline in the pool contain unlithified to weakly lithified microbial pustular sheet mats, which erode to release irregular peloidal grains. In addition, over 20 km^2^ of gelatinous microbial mats, with thin brittle layers of micrite, colonize subtidal pavements. When these gelatinous mats erode, the micritic layers break down to form platey, micritic intraclasts with irregular boundaries. Together, the irregular micritic grains from pustular sheet mats and gelatinous pavement mats make up nearly 26% of the total sediment in the pool, plausibly producing ~ 24,000 metric tons of microbial sediment per year. As such, Hamelin Pool can be seen as a microbial carbonate factory, with construction by lithifying microbial mats forming microbialites, and erosion and degradation of weakly lithified microbial mats resulting in extensive production of sand-sized micritic sediments. Insight from these modern examples may have direct applicability for recognition of sedimentary deposits of microbial origin in the geologic record.

## Introduction

Hamelin Pool, located in Shark Bay, Western Australia is home to the world’s most extensive assemblage of living microbialites^1,2^. Microbialites are organosedimentary deposits that have accreted as a result of a benthic microbial community trapping and binding detrital sediment and/or forming the locus of mineral precipitation^3^. As the first macroscopic fossil evidence of life on the planet, microbialites have been dated to ages greater than three billion years^4–8^, making living structures, such as those in Hamelin Pool, a critical analog for interpretation of ancient structures.

Although Hamelin Pool is best known for its classic microbialite buildups, often referred to as stromatolites, a recent mapping project^2^ revealed that stromatolites buildups cover less than 2% of the total area of the pool. Weakly-lithifying microbial sheet mats in the upper intertidal zone make up ~ 3% of the total Hamelin Pool area and subtidal pavements account for ~ 9% of the Hamelin lithofacies. The bulk of Hamelin Pool, ~ 86% of the total area, consists of peloid-dominated carbonate sediments.

Throughout the geologic record, microbialites and peloids are commonly associated in depositional settings. Some examples include a Late Neoproterozoic age formation in the Mackenzie Mountains of northwestern Canada, where cap carbonates, peloidal grains, and stromatolites are intimately associated^9^; a late Proterozoic-early Cambrian age formation in Namibia, where stromatolites and thrombolites are in close association with peloid grainstone facies^10–12^; a Cambrian age formation in the Great Basin in California/Nevada, where thrombolites are found intermingled with peloid-rich grainstones^13,14^ (Supplemental Fig. S1); a Devonian age formation in the Canning Basin where stromatolites and peloidal limestones commonly occur together^15^; and a Lower Cretaceous formation, where microbialite-peloid associations are common in both the Campos and Kwanza Basins^16,17^. Although a microbial origin for stromatolites and other microbial buildups in these ancient examples is well established^18–21^, sources of the peloidal sediments are mostly not discussed.

Results from our recent mapping studies suggest that Hamelin Pool may be an ideal modern analog for microbialite-peloid associations that are prevalent throughout Earth history. In this paper, we explore the role of benthic microbial communities as a prolific modern-day carbonate factory. In particular, we describe extensive microbial carbonate sediments within Hamelin Pool produced through a previously little-studied erosional process that has led to the accumulation of sand-sized micritic grains that are associated with synchronous construction of stromatolites in this world famous setting.

## Background

**Figure 1:**
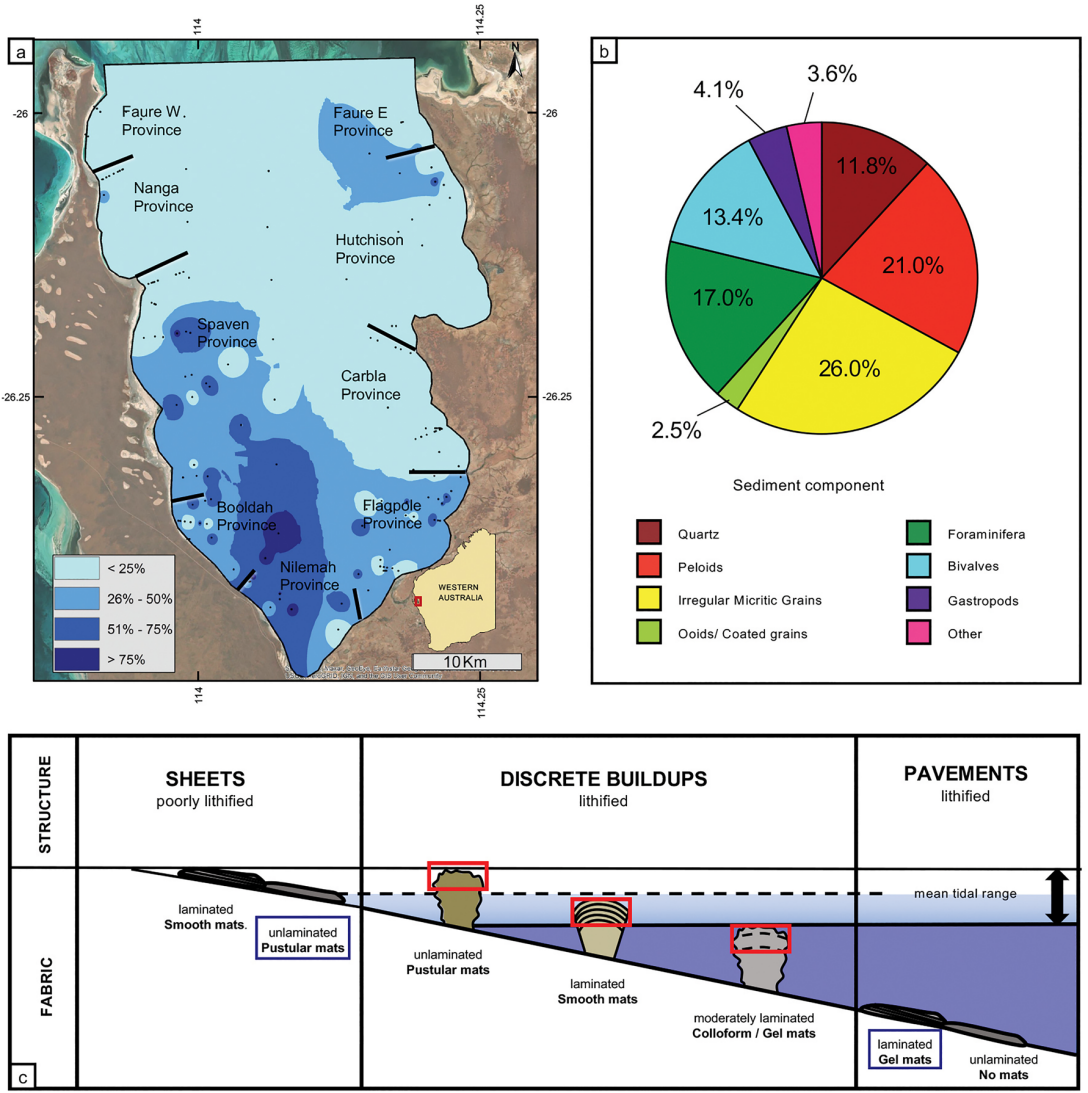
(**a**) Hamelin Pool map showing the Provinces of Hamelin Pool, the location of collected sediment samples, and the percentage of irregular micritic grains in collected sediment samples (see Supplemental Fig. S1 for comparison to peloid percentage as shown in Fig. 10c in^2^), basemap sources: Esri, DigitalGlobe, GeoEye, i-cubed, USDA FSA, USGS, AEX, Getmapping, Aerogrid, IGN, IGP, swisstopo, and the GIS User Community, created in ArcMap 10.6 https://support.esri.com/en/products/desktop/arcgis-desktop/arcmap/10-6-1; (**b**) pie chart showing sediment composition of all samples collected in Hamelin Pool. Dominant components are peloids (red) and irregular micritic grains (yellow), which make up almost half of sediments; and (**c**) the classic diagram of Hamelin Pool stromatolites which make up less than 2% of the total area in Hamelin Pool, with the addition of intertidal unlithified sheet mats, which make up over 3% of the total area of Hamelin Pool, and lithified pavements, which make up 9% of the total area in Hamelin Pool (percentages taken from^2^). Diagram modified from Suosaari et al.^28^.

Hamelin Pool, located in Shark Bay, Western Australia, (Fig. 1a), is a restricted embayment about 800 km north of Perth, Western Australia. Hamelin Pool covers roughly 1400 square kilometers and has 135 km of coastline, nearly all of which is dominated by microbial mats^2^. Hypersalinity^22^, large fluctuations in temperature, and frequent subaerial exposure create a high-stress environment that is unfavorable for growth of macroalgae and other eukaryotic organisms (sensu^23–27^) allowing extensive microbial development^28^.

The classic models of Hamelin Pool recognize microbialites (historically termed ‘stromatolites’^29,30^ in a shore parallel facies band around the margin, subclassified by their surface mats, which vary by location within the tidal zone (e.g.,^1,22,29–31^). These early studies recognized ‘pustular-mat stromatolites’ in upper intertidal zones, ‘smooth-mat stromatolites’ in lower intertidal to shallow subtidal, and ‘colloform-mat stromatolites’ in subtidal zones (Fig. 1c). Recent studies by Suosaari et al.^2,28^ expanded upon this model to differentiate between lithifying microbial mats that construct microbialites and non-lithifying to weakly-lithifying sheet mats that form broad, extensive accumulations in the upper intertidal zone (Fig. 1c), commonly located in bights and embayments. Additional facies mapping by Suosaari et al.^2^ documented subtidal pavements forming 9% of the total area and commonly coated with gelatinous microbial mats, and carbonate dominated sediments that make up 86% of the total area of Hamelin Pool. Peloids and irregular micritic grains were found to be the most abundant component in Hamelin Pool sediments (Fig. 1b), and were common throughout all Provinces with the highest abundances in the southwestern region of the Pool (Nilemah, Booldah, and southern Spaven Provinces (Supplemental Fig. S2).

In the analysis of Hamelin Pool sediments published by Suosaari et al.^2^ two types of micritic grains were described as ‘peloids’: spherical grains with distinct smooth edges and angular micritic grains with irregular edges. Together these micritic grains comprise approximately half of the sediment in Hamelin Pool, and are most dominant in the southern and southeastern regions of the Pool (Supplemental Fig. S1). The spherical peloids, comprising about 21% of the sediment in Hamelin Pool, include coated grains, micritized skeletal grains, and ooids, as described in previous studies^22,32^. Irregular micritic grains, which comprise ~ 26% of the sediment in Hamelin Pool and up to 80% of sediments in the south and south east (Fig. 1a,b), are the focus of the present study. Results complement previous studies of Hamelin Pool stromatolites^1,2,28,33–35^, and document erosional and constructional sedimentary processes, which together, constitute a modern carbonate factory that produces a microbialite-peloid facies association typical of the geologic record.

## Results and discussion

### Formation of irregular micritic grains

Irregular micritic grains make up 26% of the total sediment facies in Hamelin Pool (Fig. 1b). Examination of petrographic thin sections from microbial mats forming in the upper intertidal zone as pustular sheet mats and from subtidal gel mats forming on low-relief microbial pavement shed light on the formation of these irregular micritic grains, as documented below.

#### Micritic grains from pustular sheet mats

**Figure 2:**
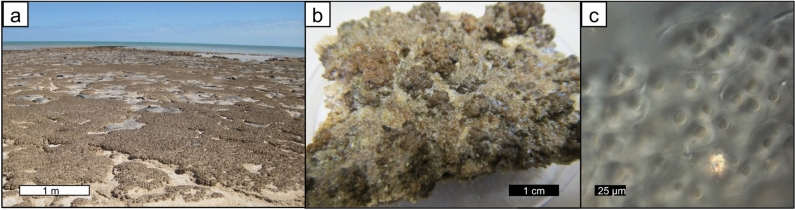
Unlithified pustular sheet mats dominated by *Entophysalis*. (**a**) Pustular sheet mats in the upper intertidal zone around the margin of Hamelin Pool in the Flagpole Province, scale bar applies to foreground; (**b**) hand sample of pustular sheet mat (HP14-JS21); (**c**) confocal image showing healthy live *Entophysalis major* cells within EPS (HP13-JS13).

**Figure 3:**
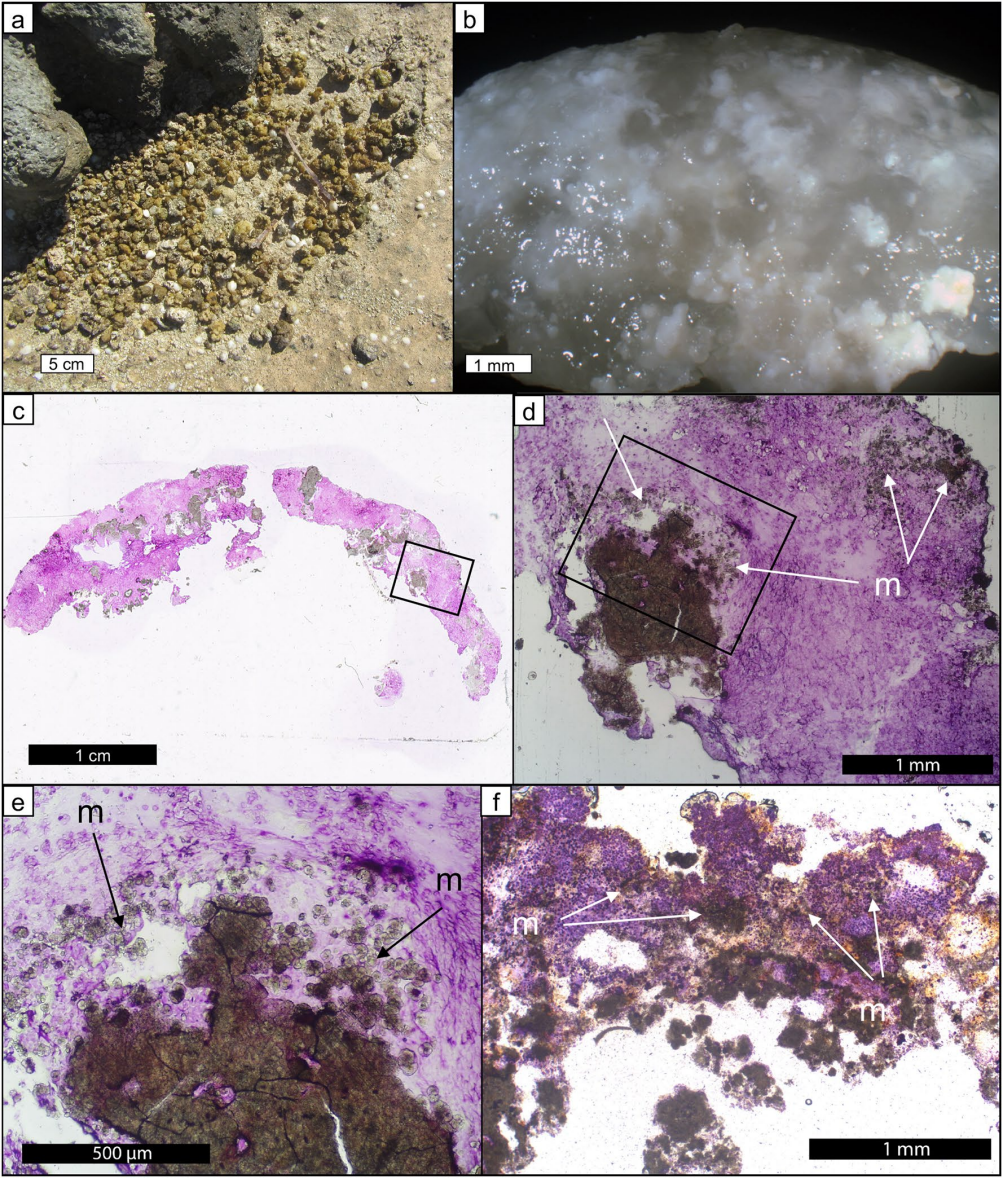
Rounded irregular micritic grain production in unlithified pustular sheet mats. (**a**) eroded pustules from pustular sheet mats; (**b**), degrading eroded *Entophysalis* pustule (HP13_T4_EP), the gel is organic matter, the white is micritic precipitate (image from Suosaari et al.^28^ supplemental material); (**c**) thin section photomicrograph of pustule shown in (**b**), *Entophysalis* cells and surrounding organics are stained purple with crystal violet, boxed area shown in higher resolution in (**d**); (**d**) *Entophysalis* cells are being replaced by microcrystalline carbonate, micrite (m, arrows), boxed area shown in higher resolution in (**e**); (**e**) *Entophysalis* cells are being replaced by microcrystalline carbonate, micrite (m, arrows); (**f**) photomicrograph of wet thin section of an eroded pustule collected after cyclone Olwyn (4_15EPS_1) showing clumps of micrite (arrows) in a matrix of *E*.* major* cells throughout the sample.

**Figure 4:**
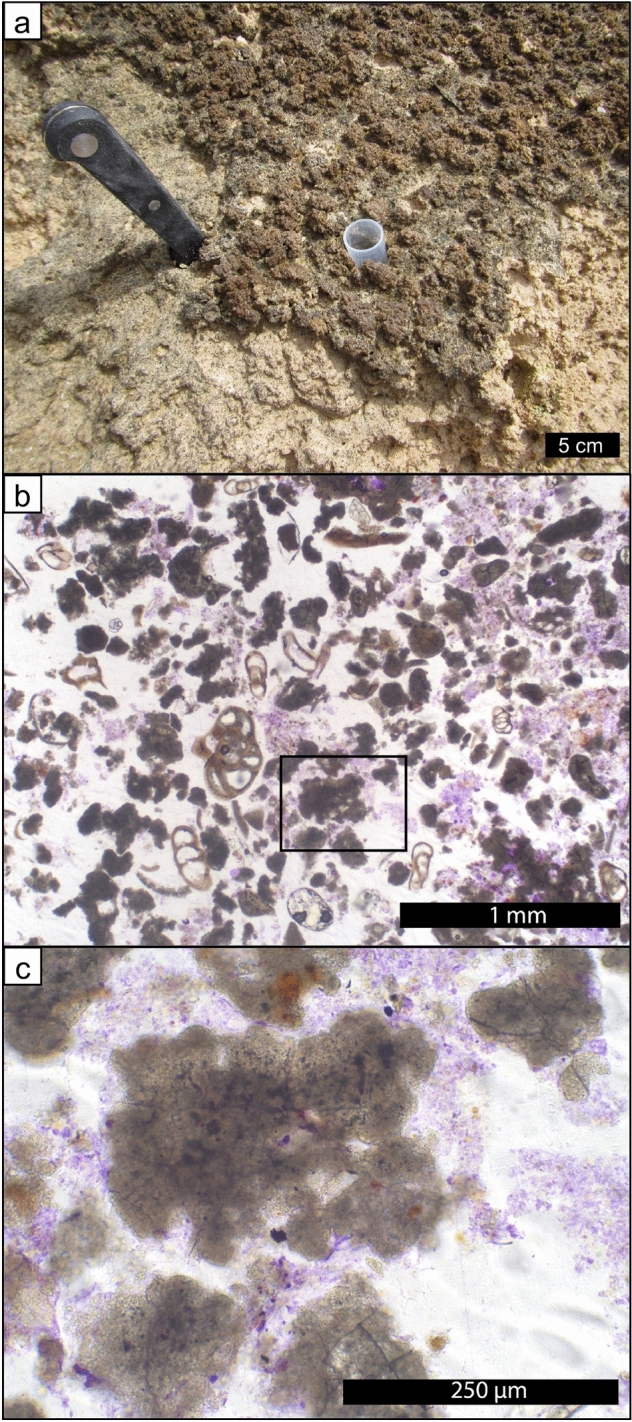
Rounded irregular micritic grain production in core beneath unlithified pustular sheet mats. (**a**) one inch diameter core taken through pustular sheet mat in Nilemah embayment; (**b**) photomicrograph of a thin section made from the core of sediment underlying the pustular sheet mat showing sediment comprised of abundant irregular micritic grains released from degrading *Entophysalis* and fresh foraminifera; and (**c**) shows a high resolution of the boxed area from (**b**) showing micritic grains formed within the pustules easily recognizable by their irregular shapes and peloidal textures.

Weakly lithified pustular mat in the upper intertidal zone of Hamelin Pool (Fig. 2a,b) is characterized by soft pustules of *Entophysalis major*, with clusters of *E*.* granulosa* and distinctive tetrads of smaller colonial coccoid cyanobacteria, all embedded within a thick matrix of exopolymeric substances (EPS) (Fig. 2c)^28^. Wet thin sections of pustular sheet mat and eroded pustules revealed an intimate relationship between *Entophysalis* and micrite (Fig. 3). The micrite originates as calcified *Entophysalis*, with dark inclusions within the micrite representing shriveled entombed cells. The mats are permineralized within the polysaccharide envelopes (glycocalyx) that surround individual and groups of cells^36–40^. Calcification of the coccoid cyanobacterium *Entophysalis* is evident in thin sections (Fig. 3d–f) stained with crystal violet, showing cells being replaced with microcrystalline carbonate and forming large clots of micrite. Thus, the soft pustules of the sheet mats are being replaced by authigenic carbonate. A one inch diameter core through pustular sheet mat (Fig. 4a) shows sediment comprised of abundant irregular micritic grains released from degrading *Entophysalis* and fresh foraminifera (Fig. 4b,c). The micritic grains formed within the pustules are easily recognized by their irregular shapes and clotted textures. Scouring wave action commonly rips up and erodes the sheet mats^22^ and pustules in various stages of decomposition are found along the edges of the pool and across the shallow sea floor (Fig. 3a).

#### Micritic grains from gelatinous pavement mats

**Figure 5:**
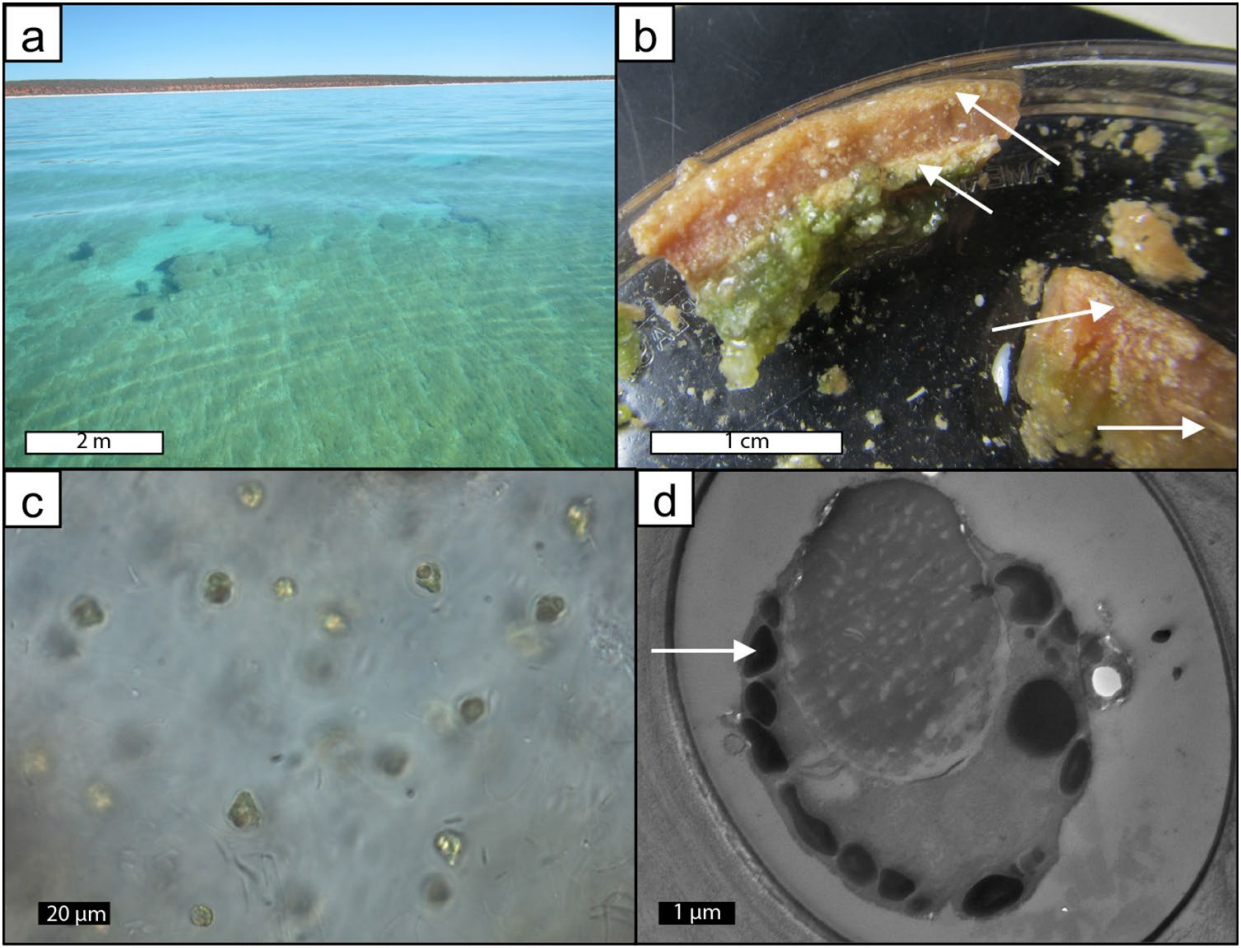
Irregular micritic grain production in gel mats colonizing the surface of low-relief microbial pavements. (**a**) Gel mats in the shallow subtidal zone of Hamelin Pool in the Booldah Province; (**b**) gel mat showing thin laminae of micritic calcium carbonate on the surface and as horizons within the mat (arrows), as well as within the mat; (**c**) phase contrast micrograph of the gel mat showing cells of the 10 µm in diameter microalga with conspicuous pyrenoid (phase bright spheres); (**d**) TEM image of ultrathin section of the microalga through the pyrenoid, revealing the starch granules (arrow).

**Figure 6:**
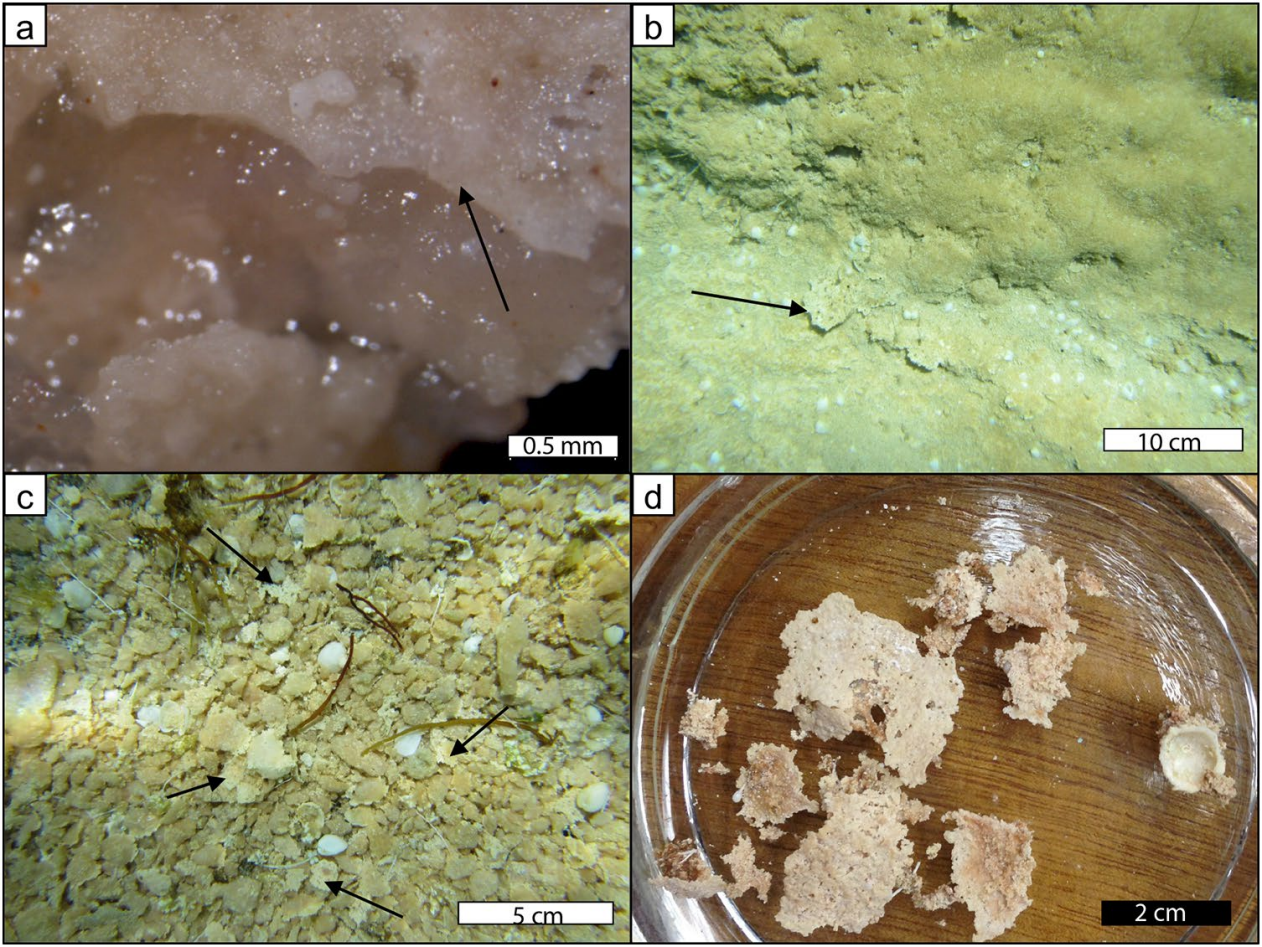
Erosion of gel mats colonizing the surface of low-relief microbial pavements showing irregular micritic grain production. (**a**) micritic crust (arrow) with gelatinous mat underneath; (**b**) platy micritic crusts (arrow) under partially eroded gel mats on top of subtidal low-relief microbial pavement in the Booldah Province; (**c**) detached, eroded globs of gel mat, some with attached crusts (arrows); (**d**) platy crusts from eroded globs of gel mat after organics were removed with bleach.

**Figure 7:**
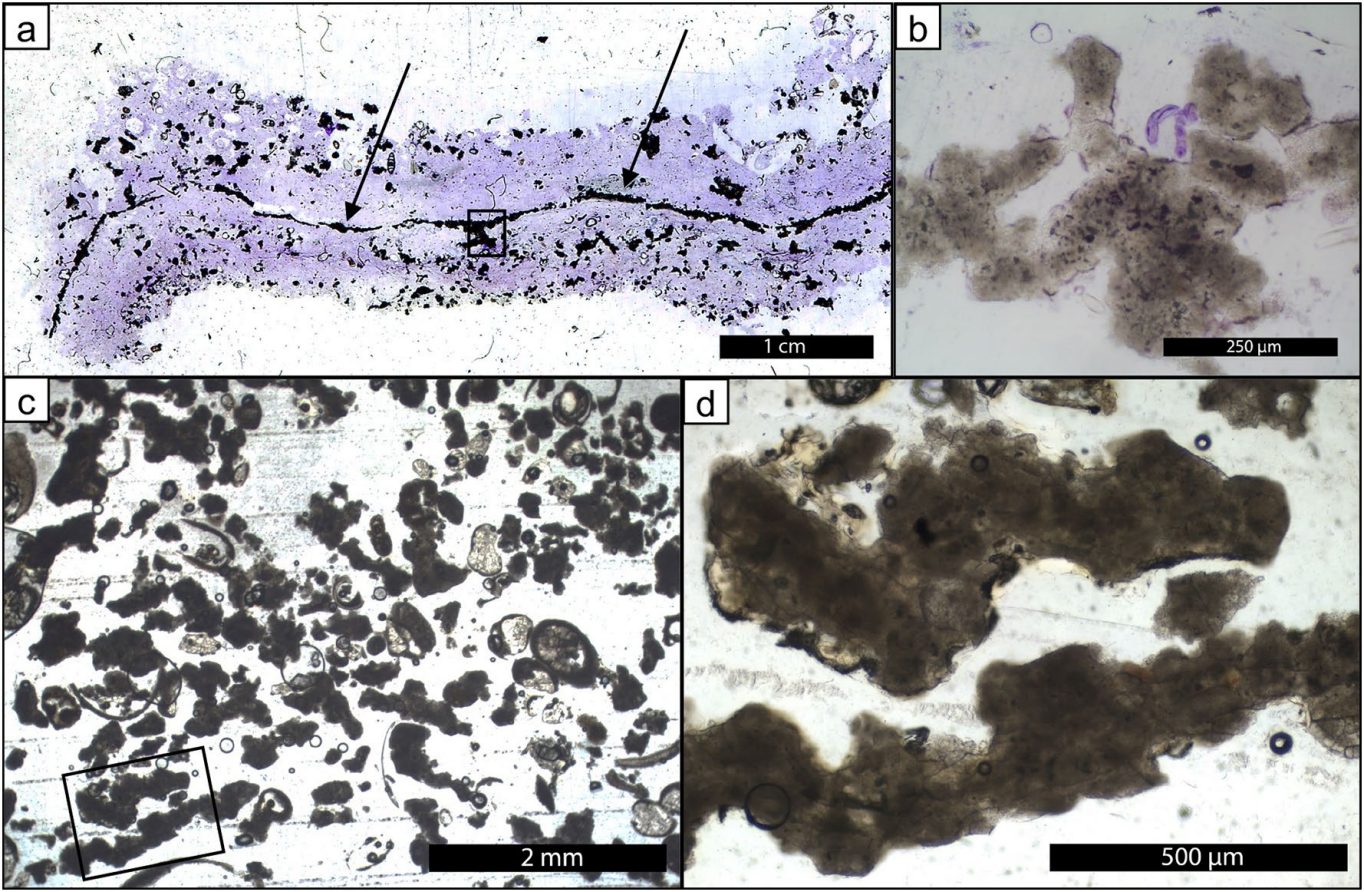
Irregular micritic grain production in gel mats. (**a**) thin section photomicrograph of showing a gel mat intersected by a micritic carbonate laminae and with homogeneous to clotted micritic textures shown in (**b**); (**c**) bulk sediment sample collected from the Booldah Province where irregular micritic grains can make up more than 75% of the total sediment; (**d**) high resolution image of micritic grains from box in (**c**) showing the characteristic irregular, often platy morphologies.

Vast expanses of gelatinous microbial mats form on extensive subtidal pavements in the southwestern region of Hamelin Pool (Fig. 5a,b). These gelatinous mats are similar in composition to the colloform and smooth mats of the stromatolites, as described by^28^, containing *Aphanothece* sp., *Aphanocapsa* sp., *Entophysalis* and diatoms. The gel mats are also characterized by microeukaryotes with pyrenoid structures (Fig. 5c,d). Thin laminae of micritic calcium carbonate are commonly found at the surface or within the gel mats (Figs. 5b, 6a). These gelatinous mats with micritic laminae break down through the degradation of organic matter, or through erosional processes in high-energy environments. Abrasion of the gel mats exposes the micritic crusts (Fig. 6b), which are subsequently eroded, forming platy fragments (Fig. 6c,d). Detached globules of gel, commonly with attached crusts, are typically seen along the southwestern margin in various stages of decomposition across the seafloor (Fig. 6c). As the gelatinous material degrades, the micritic laminae break up into platy intraclasts. These irregular micritic grains have jagged, uneven boundaries. In thin section, the micritic laminae show homogeneous to clotted micritic textures (Fig. 7a, b). In the south and southwestern regions near well-documented accumulations of gel mats on subtidal low-relief microbial pavements^2^, irregular micritic grains can make up more than 75% of the total sediment (Figs. 1b, 7c,d, Supplemental Fig. S1).

#### Preliminary estimates of production

Both pustular mats and gelatinous pavement mats appear to be prolific sources of irregular micritic grains. Rates of grain production are difficult to estimate, and growth studies of the mats have not been conducted. To get a crude estimate of grain production, we made some back-of-the-envelope calculations. Three samples of pustular mat from the southern embayment of Nilemah Province (Fig. 1a), each ~ 1 cm thick, covering areas of approximately 10 cm × 10 cm or 100 cm^2^, were dissolved in bleach. Weights of sediment released from 100 cm^2^ of each sample ranged from 5 to 10 g. This gives a carbonate production of approximately 500–1000 g per square meter of surface mat. A turnover time of 5 years for surface growth on pustular mats would give a production rate of ~ 100 to 200 g carbonate sediment per square meter of mat per year.

Similarly, 1 cm^2^ of carbonate crust from gel mat from the subtidal gelatinous mats of Booldah Province (Fig. 1a) weighs approximately 0.1 g. Thus, 1 m^2^ of gelatinous mat could contain 1000 g of carbonate crust. Again, forming a new crust every five years, would yield a production rate similar to that of the pustular mats, of about 200 g carbonate per square meter of mat per year.

These rough estimates of rates of carbonate production by weakly lithified pustular and gel mats are similar in order of magnitude to carbonate production by calcareous algae in open marine environments, which are estimated to range from 50 to 240 g m^−2^ year^−1^ in *Halimeda* and *Padina*, respectively^41^. Based on these numbers, pustular and gel mats together are estimated to contribute up to 0.4 kg m^−2^ year^−1^ of carbonate to Hamelin Pool, amounting to the addition of ~ 24,000 metric tons of microbially produced carbonate grains annually (based on 60 km^2^ of microbial mats and low-relief microbial pavements mapped by^2^.

### Microbial carbonate factory

Benthic carbonate production systems have been termed ‘carbonate factories’^42–49^. Schlager^44^ recognized three main factories: the tropical factory, the cool water factory and the mud mound factory (Fig. 4a). We here propose a modification to the Schlager factory subdivision, suggesting a category of ‘microbial factory’, adding a subdivision of ‘microbialite-peloid factory’ to the previously defined ‘mud mound’ category. Rationale for this addition is based on our observations from Hamelin Pool, applied to the geologic record, as discussed below.

#### Hamelin Pool as a modern analog

Hamelin Pool can be considered as a modern analog for the microbialite-peloid facies association that is common in the geologic record. Since the discovery of stromatolites in Hamelin Pool in 1954^30^ the constructive microbial processes that lead to formation of these microbialite structures have been extensively studied (e.g.,^1,2,22,28–30,33–35,38,50–54^ etc.). The microbial surface mats that generate the underlying stromatolite structures produce exopolymeric substances (EPS), which enhance stability^55^ and promote micrite precipitation, leading to the upward growth of the structures^56,57^. In addition to trapping and binding of carbonate sands by the microbial mats, stromatolite accretion in Hamelin Pool is accompanied by pervasive precipitation of microbial micrite^28,33,54^, often composing up to 85% of the internal fabrics^54^. Integrated studies of surface mats and internal fabrics have indicated that the microbial composition of the surface mats is correlated to the microbialite microstructures^28^.

In addition to the construction of microbialite structures by lithifying microbial mats, the discussion of micritic sediments above, as illustrated in Figs. 2, 3, 4, 5, 6 and 7, indicates that erosion and degradation of unlithified microbial mats in Hamelin Pool leads to the extensive production and accumulation of peloids—sand-sized irregular micritic grains. In the upper intertidal zone around the margin of Hamelin Pool, nearly 40 km^2^ of weakly lithified microbial pustular sheet (~ 3% total area of Hamelin Pool^2^), produce copious amounts of micritic carbonate grains through the degradation and lithification of *Entophysalis* cells. When these pustules erode and decay, the calcified microbes make a substantial contribution to the sediments in the form of irregular micritic grains. In addition, over 20 km^2^ of gelatinous microbial mats with thin brittle layers of micrite colonize subtidal pavements (~ 1.5% total area of Hamelin Pool^2^). When these mats erode and decay, the micritic layers break down to form micritic intraclasts. Together, these irregular micritic grains are the most common sediment component in Hamelin Pool, making up nearly 26% of the sediments, followed by well-rounded peloids (~ 21%), Foraminifera (~ 17%), the bivalve *Fragum erugatum* (~ 13.4%), quartz grains (~ 11.8%), followed by gastropods, ooids/coated grains, and other sediments (~ 10%) (Fig. 1b).

**Figure 8:**
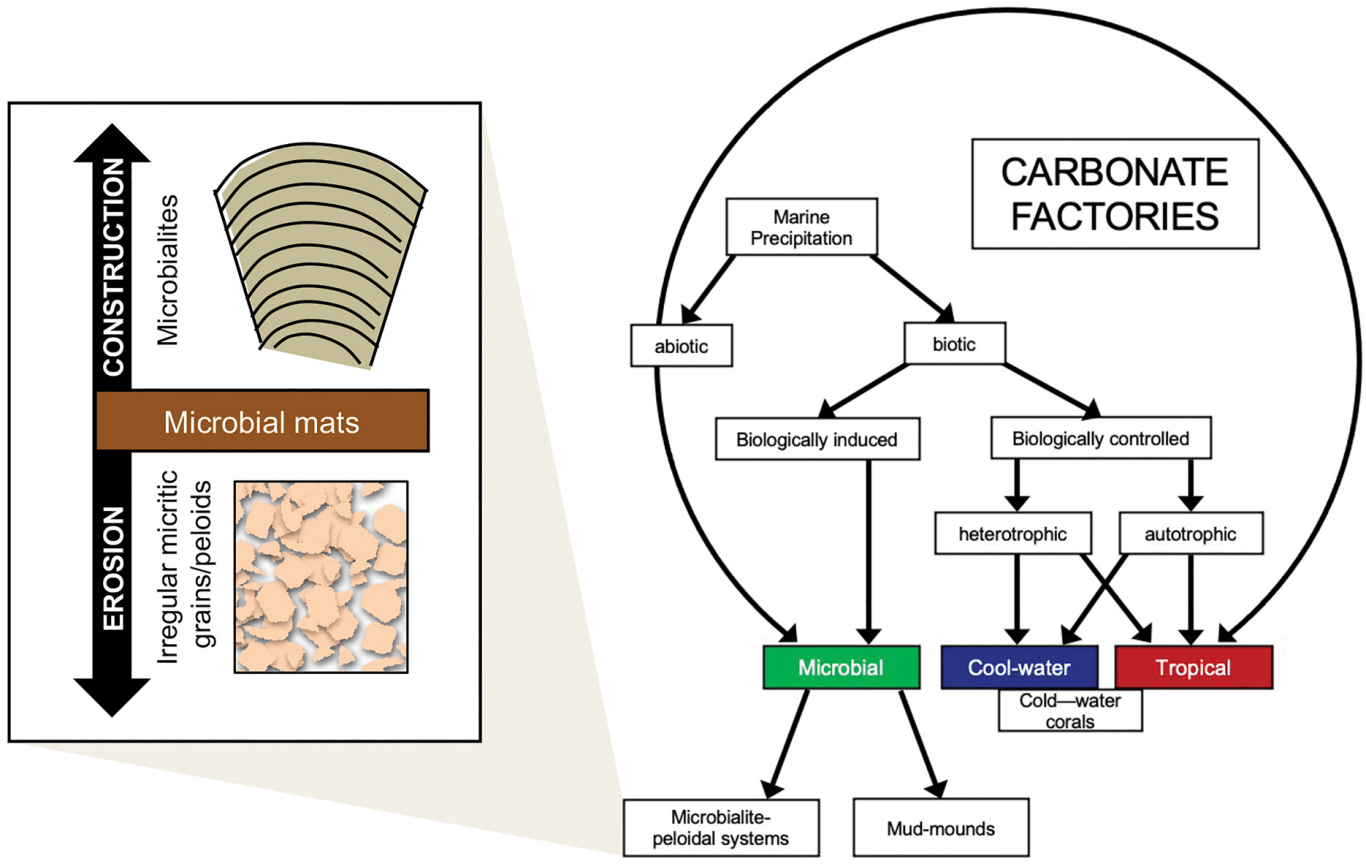
Modification of the Schlager^44^ and Reijmer^48^ carbonate factory diagram with the inclusion of a microbial pathway informed by the microbialite-peloidal system observed in Hamelin Pool with construction by lithifying microbial mats forming microbialite buildups and erosion of unlithified microbial mats forming sand-sized micritic sediments.

As such, Hamelin Pool is an effective microbial carbonate factory, with construction by lithifying microbial mats forming microbialites and erosion and degradation of weakly lithified microbial mats simultaneously resulting in extensive production of sand-sized micritic sediments (Fig. 8). Platform morphologies and sediment production rates associated with the microbialite-peloid carbonate factory are shown in Supplemental Fig. S3, updating the diagrams of Reijmer^48^ and Schlager^44^. Recognition of a microbial carbonate factory in Hamelin Pool provides a basis for interpretation of the common association of microbialites and peloids in the geologic record.

#### Relevance to the geologic record

Understanding microbial processes of sedimentation in modern environments, as described above for Hamelin Pool, helps to interpret the geologic record. In Hamelin Pool, the dominant lithofaces are sediments (86%), predominantly composed of irregular micritic grains as the dominant sediment component, whereas the microbialite facies covers a much smaller area of the Pool (< 2%)^2^. This combination of abundant peloidal sediments and relatively low abundances of microbialite structures is a common association throughout the rock record^9,13–17,58–60^; etc. The findings of this study may also provide insight into the origin of peloids in the geologic record that are not found in association with microbialites, but are not identifiable as fecal pellets or other detrital grains derived from different and/or more distal sources (sensu^61^).

In addition to the recognition of microbial activity as an important source of peloidal grains, the identification of the type of microbe contributing to the formation of these micritic grains is significant. Of particular relevance are calcifiying *Entophysalis,* and an abundant microalga with pyrenoids. For the latter, it had been proposed that 10 μm microfossils preserved in the Belcher and Bitter Springs formations might contain pyrenoids^62^. Not only are the microfossils in both the Belcher Supergroup (~ 2.0 Ga formation^63^ located in Hudson Bay, Canada), and the Bitter Springs (~ 800 Ma formation^64^ located in Central Australia) similar in appearance, both have facies associations consisting of stratiform and domal stromatolites, with accumulations of peloids, intraclasts, and/or siliciclastic grains between structures^18,62,65–68^. Similar to many stromatolites in Hamelin Pool, which display micritic frameworks^28,33^, stromatolites in the Belcher Supergroup are attributed to in situ permineralization of microbial mats rather than trapping and binding^18^. Significantly, an important bacterial microfossil identified in both the Belcher and Bitter Springs formations is *Eoentophysalis* sp.^18,69^, which is a probable precursor to modern *Entophysalis* sp.*,* the microbe contributing to the formation of Hamelin Pool stromatolites^28,36,37^, as well as and peloidal and intraclast sediments as described here (Figs. 2, 3, 4, 5, 6, 7).

In addition to the presence of *Entophysalis*, the presence of microalga with pyrenoids in Hamelin Pool subtidal gel mats is also relevant to fossil structures. Dark inclusions observed in microfossils have been interpreted as pyrenoid structures and used as criteria to identify eukaryotic cells^62^. Although these dark inclusions are present in microfossils observed in both the Bitter Springs formation and Belcher Supergroup, they have been interpreted by some authors as degradational features of prokaryotes, such as *Entophysalis*^18^. In contrast, other studies suggested that these organelle-like bodies are comparable to pyrenoids which occur in modern eukaryotic algae^62^ such as those observed in the intraclast-producing gel mats of Hamelin Pool. Our observations support the potential presence of eukaryotic algae in microbialite-peloidal systems of the Precambrian.

## Conclusions

Using Hamelin Pool as a modern analog to examine the significance and association of microbialites and peloids throughout the geologic record, we propose that microbial systems are prolific carbonate factories. Construction by lithifying microbial mats forms microbialite buildups, while erosion of weakly lithified microbial mats forms sand-sized micritic sediments, that can be lumped under the umbrella term ‘peloids’. Many Proterozoic carbonate megafacies are composed predominantly of micritic and peloidal limestones interbedded with stromatolitic structures. This microbialite-peloid association is also common in Phanerozoic carbonate ramps and platforms. Although microbial communities have long been recognized to form stromatolites and other buildups, the role of microbes in prolific production of sand-sized micritic grains has, until now, been overlooked.

## Methods

### Field studies

Fieldwork was conducted during three 2-month field seasons (March and April 2012–2014) using small boats. Samples were collected from non-lithifying sheet mats in the upper intertidal zone of the Nilemah embayment (Nilemah Province), and from gel mats on the surface of low-relief microbial pavement in the shallow subtidal zone along the southeast margin (Booldah Province). Sediment samples were collected throughout the pool.

### Sediment analysis

Point counting was conducted on photomicrographs of 152 thin sections of unconsolidated sediments from Hamelin Pool taken using a petrographic microscope in plane-polarized and cross-polarized light. Photomicrographs were visualized in JMicroVision Image Analysis Toolbox 1.2.7, and 300 randomly distributed points were counted on each slide. Results from this data set were originally reported in Suosaari et al.^2^ using seven categories including: quartz, peloids and/or intraclasts, ooids and coated grains, bivalves, gastropods, foraminifera, and other. This study focuses on only the peloid data collected from that previous report and investigates the peloids and intraclasts independently.

### Microbial mat analysis

Samples were collected and maintained in Hamelin Pool seawater for immediate microscope analysis, with a subset preserved on site with 2.5% glutaraldehyde or 4% formalin in filtered seawater. Preserved samples were kept chilled and in the dark. Light micrographs were taken on an Olympus BX51 fluorescence microscope with a Micropublisher Camera (Q Imaging, Surry BC)^70^. Microbial mat samples used in this study included pustular mat collected from a sheet mat located in the intertidal zone in the Nilemah Province, and a gel mat collected from the surface of a low-relief microbial pavement in the subtidal zone in the Booldah Province. Microbial mat samples were embedded in epoxy following Nye et al.^71^ which preserves both biological and mineral material, and the embedded material was made into petrographic thin sections. These sections were stained with crystal violet to highlight organics and examined using a petrographic microscope. For analysis using transmission electron microscopy (TEM), microbial mat samples were prepared using a modification of the method in 68 from samples stored in 2.5% glutaraldehyde. Once in the lab, the samples were cut into smaller pieces (~ 1 mm cubes) and initially rinsed in filtered seawater, then post-fixed for 1 h with 2% osmium tetroxide in 0.5 M sodium acetate. Following 3 × rinse with 0.5 M sodium acetate buffer, the mat samples were incubated overnight in an aqueous solution of 0.5% uranyl acetate. The samples were then dehydrated in an ethanol series (50, 70, 90, 95, and 100%), followed by propylene oxide treatment (first straight, then 1:1 with Spurr’s low viscosity embedding medium) and embedded in Spurr's. Ultrathin sections were stained with uranyl acetate and lead citrate (1%) and observed on a JEOL 1210 TEM (JEOL, Peabody MA) at 80 kV equipped with an ORCA HR digital camera (Hamamatsu, Bridgewater NJ).

## Supplementary Information

Supplementary Information.
