# Supplementary material for: The microbial carbonate factory of Hamelin Pool, Shark Bay, Western Australia
Source: Sci Rep. 2022 Jul 28;12:12902. doi: 10.1038/s41598-022-16651-z (PMC9334266; doi:10.1038/s41598-022-16651-z)
Supplement: Supplementary file 1 — Supplementary Information. [file 41598_2022_16651_MOESM1_ESM.docx]

**Supplemental File**

**Hamelin Pool, Shark Bay, Western Australia: A microbial carbonate factory**

Erica P. Suosaari^1,2,3,*^, R. Pamela Reid^2^, Christophe Mercadier^4^, Brooke E. Vitek^2^, Amanda M. Oehlert^2^, John F. Stolz^5^, Paige E. Giusfredi^2^, Gregor P. Eberli^2^

^1^Department of Mineral Sciences, National Museum of Natural History, Smithsonian Institution, Washington DC, 20560, USA

^2^Rosenstiel School of Marine and Atmospheric Science, University of Miami, Miami, Florida 33149, USA

^3^Bush Heritage Australia, 395 Collins St., Melbourne, Victoria, 3000, Australia

^4^Retired from Shell International, France

^5^Department of Biological Sciences, Duquesne University, Pittsburgh, Pennsylvania, 15282, USA


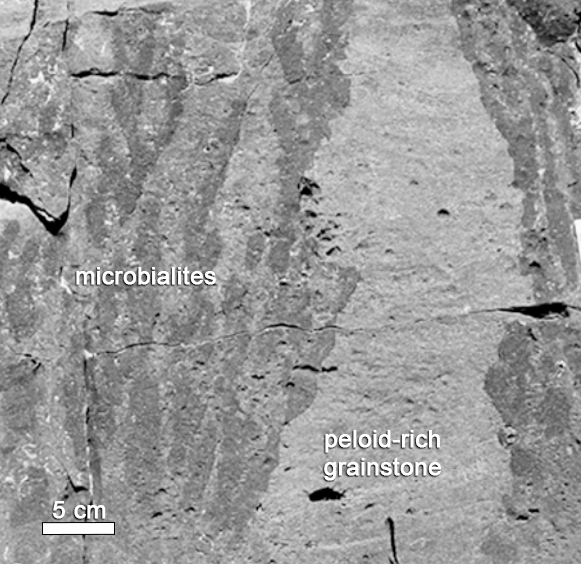


Supplemental Figure S1: Field photograph of a vertical outcrop exposure in the Great Basin, USA, Late Cambrian *Suakia* trilobite Zone showing peloidal sediments and microbialite structures, modified from Shapiro and Awramik (2006).


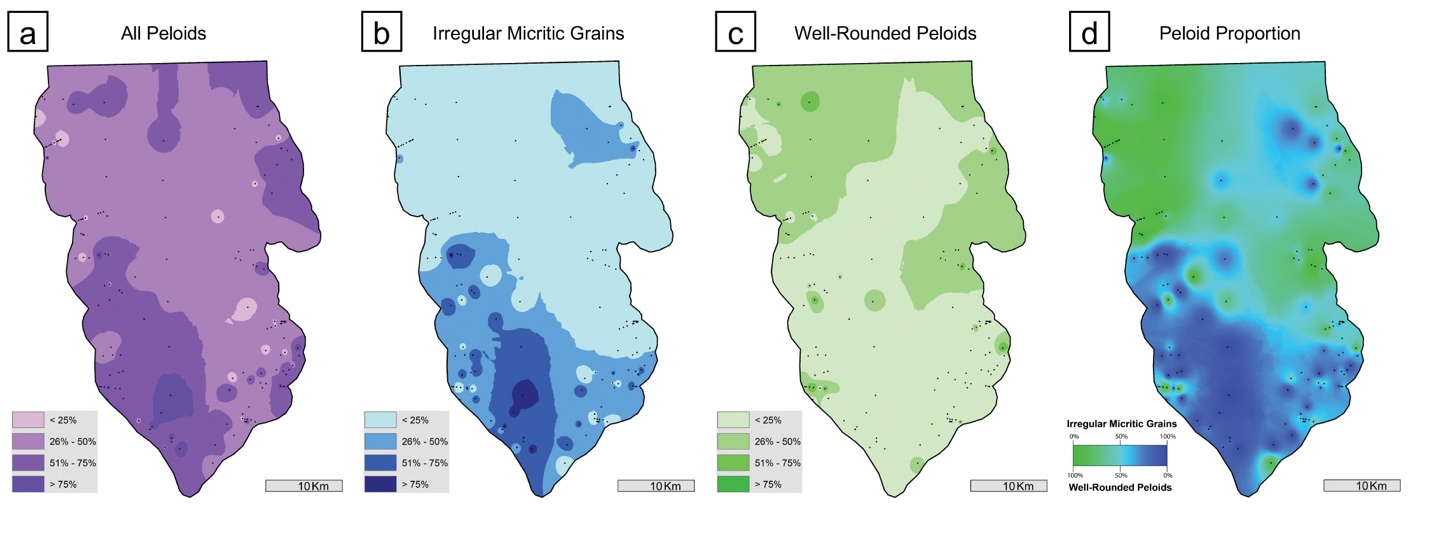


Supplemental Figure S2: Hamelin Pool map showing the distribution and % abundance of peloid sediments in of Hamelin Pool mapped using IDW in ArcGIS. 152 sediment samples, collection locations shown as black dots on the map, were made into thin petrographic thin sections and point counted (see methods section in Suosaari et al. (2019a)). (a) map showing the abundance and distribution of all peloids counted as shown in Fig. 10c in Suosaari et al. (2019a) where well-rounded peloids and irregular micritic grains were counted together, (b) map showing the abundance and distribution of irregular micritic grains extracted from the peloid dataset, (c) map showing just the abundance and distribution of well-rounded peloids extracted from the peloid data set, and (d) showing the distribution of the irregular micritic grains (blue) vs. well-rounded peloids (green) within the peloid dataset, showing that the irregular micritic grains dominate the south-southwestern region of Hamelin Pool, whereas the well-rounded peloids are more abundant in the northern region of Hamelin Pool.


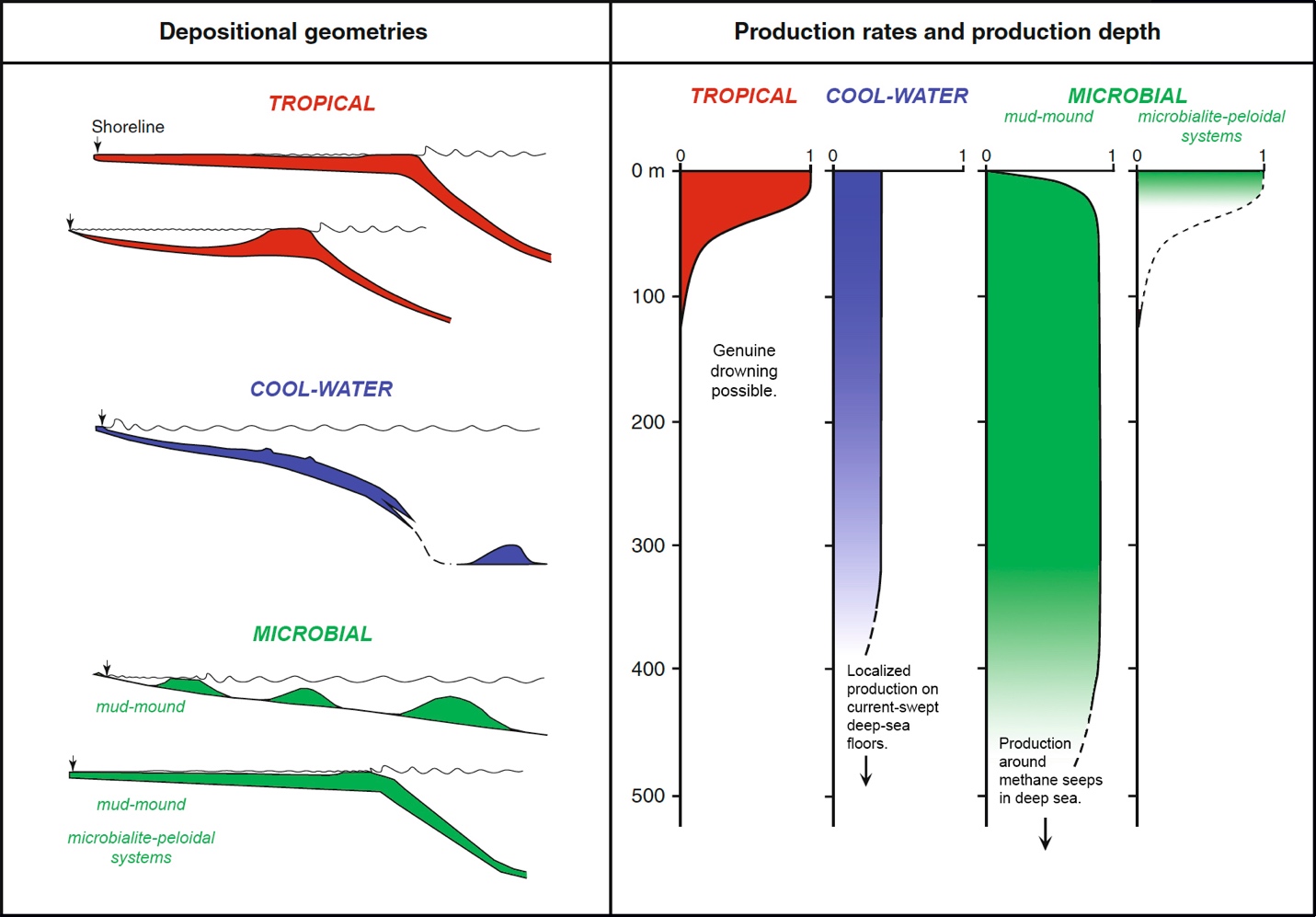


Supplemental Figure S3: Carbonate factories showing platform morphologies and sediment production rates as depicted by the Reijmer (2014) and Schlager (2003), modified to include the microbialite-peloidal system. We propose that the microbialite-peloid factory is similar to the tropical factory both in platform morphology and sediment production rates, serving as a precursor to modern coral reef systems. The accumulation geometry of the microbialite-peloid factory reflects the flat platforms, sharp shelf breaks and steep slopes that are typical of reef systems. Dominance of phototrophic cyanobacteria in the microbialite-peloid factory leads to high production rates in a narrow depth window with poorly known lower limits of production.
